# Construction of School-Enterprise Cooperation Practice Teaching System under the Big Data Internet of Things Industry Collaborative Innovation Platform

**DOI:** 10.1155/2022/2072434

**Published:** 2022-09-15

**Authors:** Daowang Li

**Affiliations:** Shandong Management University, Jinan, Shandong 250357, China

## Abstract

The school-enterprise cooperation practice teaching system has increasingly become the core of the field of education research on the basis of the Internet and mobile communications. Based on the big data IoT theory and relying on the industrial collaborative innovation platform, this study designs and implements a general service platform for school-enterprise cooperation practice teaching for IoT applications. The platform is divided into two parts: the IoT data transmission part and the Web practice teaching service section. On the Internet of Things big data transmission platform, after the big data processing provided by the intelligent industry collaborative innovation end is completed, it disguises as a web practical teaching service platform built by the Internet of things framework based on B/S architecture. It solves the problem of data transmission on the order of millions. During the simulation process, the platform realizes flexible deployment and automatic integration of online upgrades. The code management script module based on scripts such as SQL, Python, and shell can complete the online platform. The automatic upgrade finally achieves the goals of the platform being easy to maintain, simple to deploy, and flexible in business logic. The experimental results show that starting from the actual situation of a digital intelligent collaborative innovation platform in a university, the practical teaching system designed and implemented adopts the object-oriented development method, and has a three-tier architecture of the collaborative innovation platform. In addition, the functional modularization and standardization reach 88.7% and 79.4%, respectively, effectively improving the performance of the practical teaching system.

## 1. Introduction

With the rapid development of colleges and universities and the continuous advancement of college education reform, the construction of digital campuses has become an important work content of college informatization construction [1–4]. The IoT big data industry has a good foundation and faces a rare opportunity for development, but it is a very difficult and serious problem without a universal and open service platform. The circulation of big data resources is difficult, the management ability is not strong, and the value of data is difficult to be realized to the maximum [5]. The existing popular big data service platforms for the Internet of Things applications are not innovative enough in technology and weak in data support. There is a large gap [5–7]. Although the application of the Internet of Things in various fields is becoming more and more extensive, there have been many Internet of Things business platforms and application cases in different industries, but the platform system is not open enough, the application development cycle is long, the compatibility of equipment access is poor, and data service accidents due to a series of reasons such as frequent occurrence, the fragmentation of the market is relatively serious.

The development of mobile communication technology and artificial intelligence digital technology provides a basic technical environment and favorable conditions for the application of Internet of Things technology in colleges and universities. The early Internet of Things technology was mainly used in the logistics industry, but now with the rapid development of information technology, the application scope of the Internet of Things is more extensive [8–10]. In recent years, the application field has gradually radiated from the logistics industry to the fields of smart home, community monitoring, video monitoring, remote assistance, environmental monitoring, biomedicine, and intelligent infrastructure. Therefore, based on studying the related architecture and digital campus of the Internet of Things, the basic ideas and system plans for building a digital campus of colleges and universities based on the Internet of Things are proposed [11–13].

Taking the digital information construction of a higher vocational college as an example, this study discusses the design idea of the digital campus based on big data and the Internet of Things. Taking the digital collaborative innovation platform as an example, the problems and solutions in the digital construction are studied and implemented in a digital information management system. The key technologies of big data Internet of Things and the problems and solutions faced in their application are studied, and the application entry point and existing solutions of big data Internet of Things in the digital campus are introduced in detail. The design scheme and model of the digital intelligent library system are introduced, and the specific book lending business process and the application of the future big data Internet of Things technology in the collaborative innovation platform are given. The common methods of campus unified identity verification are studied: password-based verification, smart card-based verification, digital certificate-based verification, and face-recognition-based authentication, and select the appropriate authentication method for the digital campus system. Combined with the actual situation of colleges and universities, a digital campus verification platform for colleges and universities based on the Internet of Things technology is proposed. In this study, the network-based three-dimensional practice teaching network database system is researched deeply, and the three-dimensional practice teaching network database system is designed and implemented. The system can provide services for teaching production enterprises, and can provide good data support for the teaching design and production of enterprises.

## 2. Related Work

The perception layer of the Internet of Things mainly realizes intelligent perception functions such as information collection, capture, object identification, and identification. Its key technologies include big data Internet of Things technology, sensors, short-range wireless communications, cloud computing networks, grid computing, and self-organizing networks. When the key is pressed, it means that an interrupt request is sent to the MCU. If the MCU turns on the external interrupt, it enters the interrupt service program. After entering the interrupt service program, the MCU needs to detect and determine which group of keys are pressed [14–16].

Li et al. [17] believed that synergy is the physical joint effect formed by two or more subsystems or elements existing in the social system through mutual dependence, and pointed out that the maximum utilization of the technology, knowledge, and other resources owned by the enterprise is collaborative innovation key. They found that different collaborative innovation models have different results for the technological innovation process of enterprises. Based on the theory of open innovation, Zhang [18] believed that enterprises should not only achieve collaboration from the internal innovation resource system, but also the external innovation system. Liu and Pingxiu [19] believed that collaborative innovation is a complex dynamic process based on the realization of a unified goal in the process of technological innovation, through interaction and cooperation among participants, and finally realizing the goal. From the perspective of forming key nodes of collaborative innovation network, Meng [20] pointed out that colleges and universities, scientific research institutes, enterprises and intermediary organizations constitute the key nodes of the SME innovation network. Based on the impact of bounded rationality, information asymmetry, and other issues on the school-enterprise collaborative innovation system, this study empirically tests the dynamic mechanism model of the school-enterprise collaborative innovation system.

The researchers measure the risk degree of the school-enterprise collaborative innovation system through the effective combination of the Monte Carlo simulation method and risk matrix [21]. In the theory of regional collaborative innovation, it is believed that the industrial innovation model tends to be the agglomeration of innovation resources, the parallelization of innovation links, and the creative integration of technology, organizational system, and organizational culture. The researchers take high-tech industries and general manufacturing industries as research objects, through the comparative analysis of collaborative innovation mode and realization path, it is concluded that the best mode of collaborative innovation development is technological innovation. From the perspective of synergy, scholars have analyzed the main factors affecting the agglomeration of high-tech industries, including market demand, location endowment, economic level, and industrial characteristics. Only in this way can the value of innovation resources be maximized, so as to carry out heavy optimization and in-depth cooperation, thereby improving their respective innovation levels [22–24].

## 3. Big Data IoT Industry Collaborative Innovation Platform System

### 3.1. IoT Topology Dependencies

The network layer of the Internet of Things is mainly responsible for information communication, which includes the access layer and the core layer. The network layer can rely on the public telecommunication network, mobile communication network, campus wireless local area network and the Internet, and can also rely on the professional communication network in the industry. The application layer of the Internet of Things mainly includes various applications, such as environmental monitoring services, intelligent communication, intelligent home, and public safety with a clean Python environment, for example, environment A has IoT installed, and environment B has IoT installed. If there is no virtualenv, you need to manage the paths of these packages yourself.

(1) $$\begin{array}{c} t\left(estermate,i,j\right)=\left\{\begin{array}{c} m\left(c\left(i\right)di\right),\\ n\left(c\left(i\right)di\right),\\ w\left(c\left(i\right)di\right). \end{array}\right.. \end{array}$$

The implementation of virtualenv is also very simple, which is to copy a complete Python environment to a separate directory and set several environment variables. Once you enter a certain env, all operations are carried out in this directory and will not affect other env environments. So virtualenv is a necessary weapon for development. There is an additional in front of the prompt. After running python, you can see that sys.path has been changed to various packages in the/srv/vpython/shici directory, run easy_install to install the required packages. Now the installed packages will all be installed into the shici environment, without affecting the system Python environment and other env environments. After installing the required packages, exit the virtual environment with the deactivate command.

**Figure 1:**
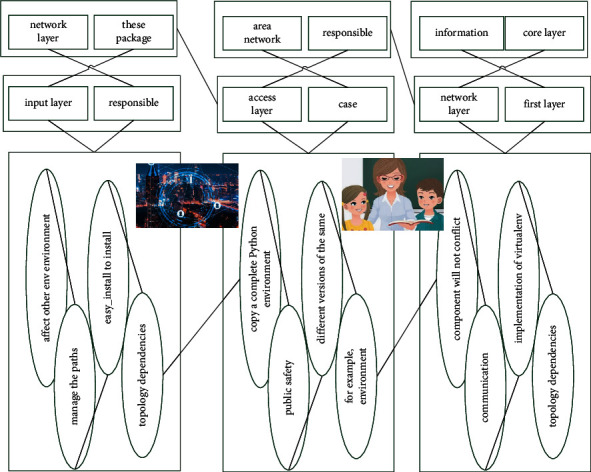
Topological level of practical teaching under the internet of things.

The format in Figure 1 is the basis of size extraction in the BMS system. All extract actions performed by the BMS software must be performed based on this 3D practical teaching network model. Other data formats cannot complete the process of size extraction.

(2) $$\begin{array}{c} \left\{\begin{array}{c} b-\frac{bw\left(n\right)}{n\left(a+b+v\left(a\right)/v\left(b\right)-1\right)}=1\\ \frac{aw\left(n\right)}{n\left(a-b+v\left(a\right)-1\right)}-1=0. \end{array}\right. \end{array}$$

Therefore, the csv file plays a very important role in the BMS system. Like the picture information (tif file), it can be considered the basic data. Unfortunately, this format does not support interactive functionality, i.e., it is not possible to do anything useful with it without the BMS software. RMSE represents the sample standard deviation of the difference between the predicted value and the observed value, that is, the prediction error. The smaller the value, the more accurate the results of the prediction model.

### 3.2. Big Data Coding and Reading

In order to ensure the uniqueness of the signed big data encoding private key, it cannot be backed up and archived. If it is lost, it is only necessary to regenerate a new key pair, and the original signature can be backed up by the old public key to verify.

(3) $$\begin{array}{c} \frac{\sum_{i+j=1}n\left(x,y\right)m\left(x,y\right)-n\left(x\right)/n\left(y\right)-m\left(x\right)/m\left(y\right)}{\sum n\left(i,j\right)-m\left(i,j\right)-1}=1. \end{array}$$

**Figure 2:**
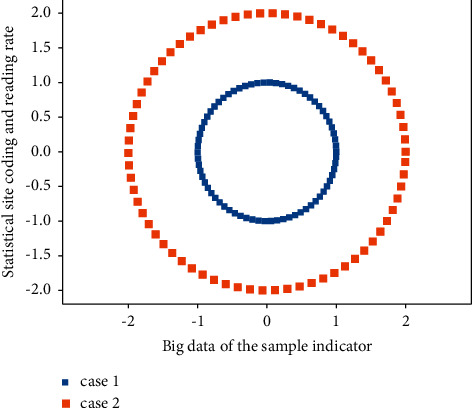
Network distribution of big data coding and reading.

The pair of keys used for digital signatures can generally have a longer lifetime. An encryption key pair consists of an encryption public key and a decryption private key. In order to prevent the loss of data when the key is lost, the decryption private key should be backed up in advance, and the archive process of Figure 2 may also be required so that the historical ciphertext data can be decrypted at any time. The encryption public key does not need to be backed up and archived. When the encryption public key is lost, it is only necessary to regenerate the key pair.

Stdin parameter, stderr parameter, stdout parameter: stdin refers to the standard output of the program, stderr refers to the standard error of the program, and stdout refers to the standard output of the program. Valid values for the parameter stdin can be PIPE, an existing file descriptor, an existing file object, or none.

(4) $$\begin{array}{c} famer\left(pi,t\right)=\sum_{i+j=1}d\left(t,y\right)d\left(x,t\right)-n\left(pi\right)/n\left(pi\right)-\frac{m\left(x\right)/m\left(y\right)}{m\left(x\right)-m\left(y\right)}. \end{array}$$

If the parameter is set to none, it needs to inherit from the parent process; the parameter stderr can choose stdout, the file handle captured in the application and used as the standard output stream stdout is obtained from the standard error data; the parameter stdout can be PIPE, create a channel for the child process.

### 3.3. Industry Cascade List

The industrial cascade of readers determines the working frequency band of the identification system, and secondly, the power of the reader directly affects the distance of RFID. The basic composition of the reader is divided into hardware and software parts. The software part is mainly responsible for responding to the instructions received by the read head and issuing corresponding action instructions to the tag. The client application connects with the middleware server by calling the functions in the dynamic connection library of the client communication module. The client application can create multiple communication connections as needed, and each successful connection will return a connection number.

(5) $$\begin{array}{c} ∵c=1,2,3,\ldots ,n,∴N\left(\left(caster\left(c\right),c\right)\right.-\frac{caster\left(c\right)}{1-caster\left(c\right)}-\frac{1-c}{c}=1. \end{array}$$

The client application can assign a unique identification (called a terminal identification) to each running working terminal to distinguish different working terminals, and can also use this identification to verify the identity of the client.

**Figure 3:**
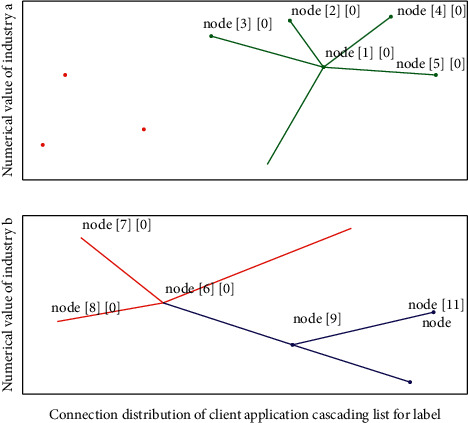
Cascading list distribution of industrial client applications.

When constructing the features in Mode 2 in Figure 3, this module will extract the features of running speed from the Map stage, the Reduce stage and the entire computing stage respectively. The features extracted in this way can be better used to describe the task running process. For each stage, this study uses the average running speed and completion percentage of this stage to describe the running state of this stage. The initial resource recommendation module will recommend to the tenant the configuration of computing resources that can complete the task on time and with the least rental cost according to the deadline and workload of the tenant's computing task.

(6) $$\begin{array}{c} M\left(\max\left(x,y\right),\min\left(x,y\right)\right)=\left\{\begin{array}{c} \max\left(x\right)-\max\left(y\right)-caster\left(c\right)/x,x.>y,\\ \max\left(x\right)-\max\left(y\right)-cx,x<y. \end{array}\right.. \end{array}$$

The big data IoT uses a multi-layer distributed application model. The application logic is divided into components according to their functions, and each application component is distributed on different machines according to its layers. The original intention of SUN to design the big data Internet of Things is to solve the drawbacks of the C/S mode. In the traditional mode, the client plays too many roles and becomes bloated, and the system's upgradeability and scalability are not high. The current multi-layer enterprise-level application model of the big data Internet of Things divides different layers in the c/s model into many layers. A multi-layer application can provide an independent layer for different services. The big data Internet of Things application model mainly consists of four layers: client layer, WEB layer, business layer, and data layer.

### 3.4. Weight Distribution of Collaborative Innovation

The collaborative innovation code information includes basic information on items such as type, name, and time, as well as reference information such as overview items and item performance. Although the information that the EPC code can store is very limited, it establishes a connection with the corresponding background database management and can obtain detailed information about the item corresponding to the EPC code through the name domain name resolution server. The Web service platform module adopts a new type of B/S architecture, and the database is installed on the server side.

(7) $$\begin{array}{c} \left\{\begin{array}{c} \left\{X,Y\subset C|p\left(i,j\right)\in X\right\},\\ no\;de\left\{i=1,2,3\ldots ,n;j=1,2,3\ldots ,n-1\right\}. \end{array}\right.. \end{array}$$

The data interaction and display are based on the browser software through the Web Server and the database system. The web server software makes one-key upgrade, installation, and update of the platform software possible through a series of script combinations. From the structural analysis of the server, in order to improve the scalability of the platform and facilitate the upgrade and maintenance of the platform, the distributed design scheme in Table 1 is adopted in the design of the big data service platform of the entire Internet of Things application.

Similar to WSGI, uWSGI is also a communication protocol. The protocol is included in the uWSGI server and uses this protocol to define the performance of the type of transmission information. uWSGI is a python web server, also known as Server/Gateway. The uWSGI server is similar to tornado web and flup. The uWSGI server completes the implementation of the uWSGI protocol and the WSGI protocol. In the process of task calculation, the multi-modal neural network of the elastic resource management module can extract the characteristics of three modalities such as the number of computing resources, the utilization rate of computing resources and the computing speed of the task, and make more accurate predictions of the task completion time in real-time.

(8) $$\begin{array}{c} \left\{\begin{array}{c} Nodes\left(1,2,\ldots ,n\right)\notin \left(1,0\right),\\ \left(n\left(i\right)-n\left(j\right)+1=0\right)=\emptyset ,\\ c=no\;de\;s\left(i,j\right). \end{array}\right.. \end{array}$$

The run-server in the Internet of Things is a simple web server. The common way to start the server is to execute the startup command through putty. Although debugging and testing are very convenient in this mode, if Putty is closed or command execution is exited, the service will stop immediately, and the service in this mode cannot withstand the load of multiple users using the service at the same time.

## 4. Construction of School-Enterprise Cooperation Practice Teaching System under the Big Data IoT Industry Collaborative Innovation Platform

### 4.1. Big Data IoT Hardware Architecture

The 3D practical teaching network data used in this article was acquired by the German TechMath non-contact 3D practical teaching network scanner. The system includes VitusSmart 3D laser practice teaching network scanning hardware system and ScanWorX digital practice teaching network automatic measurement software. The VitusSmart 3D laser practice teaching network scanning hardware system uses laser scanning to scan an area of 225 × 220 × 285 (cm) in 8–10 seconds, with a resolution of 0.5 ram. The ScanWorX digital practice teaching network automatic measurement software can process more than 500,000 data points on the practice teaching network, display the scan results in three-dimensional images, and the system automatically measures according to certain rules and reports the size of 85 parts of the practice teaching network.

(9) $$\begin{array}{c} \left[u\left(i-1,i+1\right),u\left(i-2,i\right),u\left(i-3,i-1\right),\ldots ,u\left(1,0\right)||i=1,2,3,\ldots ,n\right]↔\left[u\left(i,for,\left\{i=i-1,0\right\}\right)\right]. \end{array}$$

**Figure 4:**
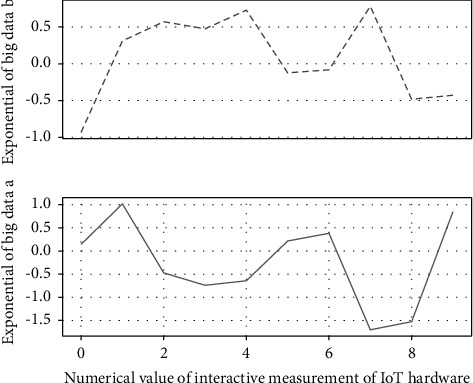
Interactive measurement of big data IoT hardware.

The system can also extract and analyze the cross-sectional data shown in Figure 4 through interactive measurement, which is used for in-depth analysis of each part of the practical teaching network.

This study focuses on analyzing each design link of the campus security management platform and constructs the overall framework of the security management system from the requirements analysis, database design, etc. Then it describes the design and implementation of the system in detail, and exemplifies part of the implementation interface. Finally, the system is tested and summarized.

(10) $$\begin{array}{c} f\left(m,n,v\left(m\right),v\left(n\right)\right)\overset{m,n<m+n}{⟶}f\left(m\right)f\left(n\right)-v\left(m\right)v\left(n\right),for\left\{m=n-1\right\}. \end{array}$$

The other features used in this model can be considered linearly related to each other and do not interfere with each other. Therefore, the functional relationship to be fitted in the prediction model can finally be obtained as node. During the loading process, the runtime library can determine whether this code has permission to be accessed, and the developer can restrict the permission of each program code. Different user login runtime also provides different security.

### 4.2. Industrial Collaborative Innovation Sequence Arrangement

The main function of Scrvlet is to receive HTRP requests from web browsers of industrial collaborative innovation clients, process input parameters, and perform operations in internal methods, such as accessing databases, accessing other Servlet methods, calling FJB, and so on, then return the processing result to the client. The result is then sent back in the form of P that the browser can display. It is similar to CGI, and PERL scripts on traditional web servers.

(11) $$\begin{array}{c} \sum_{m,n=1}1-\frac{1-n-m}{f\left(m\right)f\left(n\right)-v\left(m\right)v\left(n\right)}-trace\left(f\left(m\right),f\left(n\right)\right)-v\left(m\right)v\left(n\right). \end{array}$$

One major difference between CGI scripts and servlets is that COl scripts start a whole new process for each request—which requires extra overhead, while servlet execution requires only starting a separate thread within the servlet engine.

For the entire calculation process, this study uses the mean value of the completion time interval of the two sub-computing tasks to describe the running speed of the entire task. The smaller the value, the faster the task is completed, otherwise, the task is running slower. It can overcome the shortcomings of traditional barcodes that cannot identify single products, can only be identified by contact, and are easily damaged and lost.

**Figure 5:**
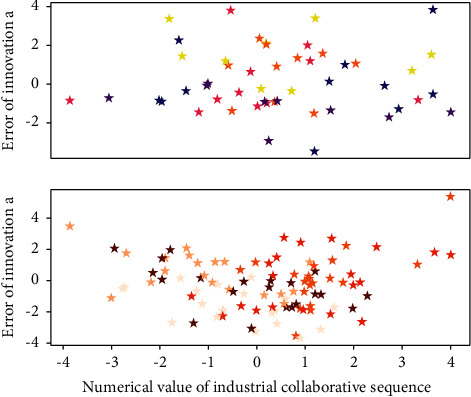
Clustering distribution of industrial collaborative innovation sequences.

The communication data frame between the intelligent terminal and the Web service platform includes three parts: the frame header, the frame trailer, and the data identification area. The frame header is used to represent the starting position of the frame data in Figure 5; the frame end is used to represent the end position of a frame of data; the data identification area mainly includes four parts of subsequent data length, identification bit, data area and check bit. After the data receiver receives the frame header of the data frame, the subsequent data received is the valid data length, and then the data receiver receives the data corresponding to the valid length according to the length, and finally realizes the complete reception of one frame of data. It mainly includes supermarket consumption management, laundry consumption, etc., for multimedia and laboratory management, including computer room management, laboratory management.

(12) $$\begin{array}{c} \sum_{m,n=1}\frac{1}{m-1}-m\sum_{m,n=1}1-\left\|\frac{1-n}{f\left(m\right)f\left(n\right)}\right\|-\frac{n}{f\left(m\right)-f\left(n\right)}. \end{array}$$

The function of the check digit in the data identification area is to accumulate the data in the data area, and then perform the accumulation result to 0 × FF, and find the remainder operation to achieve the purpose of verification. The main function of the data area is to store valid data, and the format difference of the data frame can be judged in this area.

### 4.3. The Division of Practical Teaching Levels

The practical teaching layer uses the smart card to authenticate the user's identity. When the smart card is inserted into the identification hardware device, if the identity is verified as legitimate, the random number in the smart card is sent to the AS for further authentication. This smart card-based authentication method can physically ensure the security of user information because of its hardware. That is, the information data cannot be easily forged, and the pre-stored data cannot be directly read.

The corresponding process converts the book information in the memory into XML format, and then provides the information to the client. The specific book location tracking process is described as follows: by attaching an RFID tag to each book, and setting up multiple fixed readers in the entire library's collection range.

The time and location data of the students approaching the dangerous area of the campus are transmitted to the database server in Table 2 through the network, and the student's whereabouts data in the school are recorded in real-time. At this time, the system will also immediately send a short message to the class instructor and relevant personnel to notify the students of the class violation text messages so that appropriate measures can be taken.

(13) $$\begin{array}{c} x\left(i,j=i-1\right)+x\left(i,j=i-2\right)+x\left(i,j=i-3\right)+\ldots +x\left(i,j=i-n-1\right)=1-\sum x\left(i\right)-x\left(i-1\right). \end{array}$$

The system will also provide a server web page, allowing parents to use the web page query method of a personal computer or PDA browser to understand the status of students' illegal activities in the school. Hierarchical algorithms can detect data at different levels of control, and it is easy to implement similarity measures or distance measures. However, the termination conditions of pure hierarchical clustering algorithms are ambiguous, and the operation of merging or splitting clusters is not correctable, which is likely to result in low-quality clustering results. The Internet connects all data transmission networks through TCP/IP technology, which can realize data transmission and access in a relatively short time, but there are also problems such as service quality, security, and mobility.

(14) $$\begin{array}{c} n\left(persate\left(\left(x\right)\right.,x\right)=n-\frac{1-x}{x-persate\left(x\right)}-\sqrt{persate\left(x\right)-x}*x\left(over-head\right). \end{array}$$

The effective combination of the hierarchical method and other clustering methods can form multi-stage clustering, which can improve the quality of clustering.

## 5. Application and Analysis of School-Enterprise Cooperation Practice Teaching System under the Collaborative Innovation Platform of Big Data Internet of Things Industry

### 5.1. Feature Extraction of IoT Big Data

The Internet of Things design pattern is a formal representation used by object-oriented programmers to solve programming problems. At present, in most Web applications with Browser/Server structure, the browser directly interacts with the user in the form of HTML or JSP, and responds user's request. Although intuitive, most management information systems operate on staggering amounts of data, bloating JSP pages with more code, and overloading Web servers.

(15) $$\begin{array}{c} \frac{\sqrt{persate\left(x\right)-x\left(i\right)}+\sqrt{persate\left(x\right)-x\left(j\right)}-1}{persate\left(x\right)-x\left(j\right)/persate\left(x\right)+x\left(j\right)}=1. \end{array}$$

**Figure 6:**
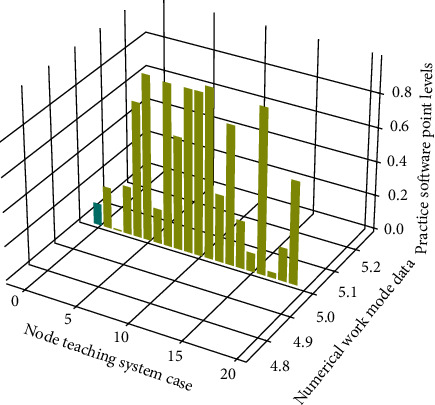
Distribution of software working mode points of practical teaching system.

Therefore, the design pattern based on Model-View-Controller (MVC) is adopted on the middle layer of Figure 6. An important implementation framework of the big data IoT idea is the MVC pattern. The design and realization of this system adopt this development mode.

Every time the IoT smart hardware sends a request, a Python dict is added to the heartbeat, for example, {“WD001”: “2018.12.01”}, WD001 in the dictionary represents the number of the smart terminal, the second half represents the date of sending the data request, the data after the transmission middle-layer platform receives the terminal's request, it refreshes the last communication time of the intelligent terminal, and when it does not receive a new request from the terminal within a certain period of time, the terminal is considered to be offline. The heartbeat data in data transmission may be highly concurrent, and the data format needs to be agreed upon between the terminal and the web server during the heartbeat data transmission process.

(16) $$\begin{array}{c} \frac{\sum \left|\sqrt{persate\left(x\right)-x\left(i\right)}\right|-\sqrt{persate\left(x\right)}}{\sum \sqrt{x\left(i\right)/x\left(i-1\right)-\sqrt{x\left(i\right)}}}=1. \end{array}$$

This design uses the Redis cache mechanism to ensure that the heartbeat data is transmitted and the MySQL database cache interaction. The data plane is mainly used to transmit and send data packets; the control plane configures the data plane to send packets to optimize data throughput and network delay to ensure sufficient reliability and stability; the knowledge plane is for control.

### 5.2. Simulation Realization of School-Enterprise Cooperation Practice Teaching System

**Figure 7:**
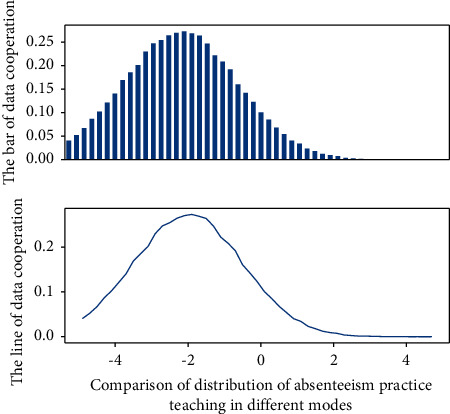
Data distribution of absenteeism in school-enterprise cooperative practice teaching.

In the practical teaching system, active big data IoT readers are deployed at school gates and important entrances and exits to actively detect students wearing active big data IoT identification tags. The time data detected by students arriving/leaving the school gate are transmitted to the school database server through the network, and the data of students' absence and absence are recorded in real-time. For students who are late or absent from school, teachers can send text messages or E-mails in real time to notify parents and relevant school administrators after confirming the unexcused absence of students. The system will also provide a server web page, allowing parents to use the personal computer or PDA browser web page query to understand the attendance status of Figure 7.

The digital intelligent sensor is an important development trend, and embedded intelligent technology is an important means, which is characterized by the combination of hardware and software. At the same time, it is combined with artificial intelligence technology, relying on existing Internet and mobile communication technology, and comprehensively using grid computing platforms and cloud computing technology to promote the realization of intelligent sensor technology in the intelligent environment of the Internet of Things.

(17) $$\begin{array}{c} \sum \sum \sqrt{p\left(i\right)/x\left(i-1\right)}-\sqrt{x\left(i\right)/p\left(i-1\right)}=\langle \begin{array}{c} 1-p\left(i-1\right)\\ 1-p\left(i\right)/x\left(i-1\right). \end{array}. \end{array}$$

Client security always occupies the most critical position in the construction and maintenance of the entire database security. Screening and filtering all kinds of resource information that may endanger the security of the database not only prevents the intrusion of the database from illegal access customers but also ensures the security of the data and information that the database ultimately transmits to the visiting customers. Data transmission refers to the process of information data from leaving the storage center to reaching the client terminal. During this process, information is in high-speed operation, and it is easy to be monitored or illegally stolen.

**Figure 8:**
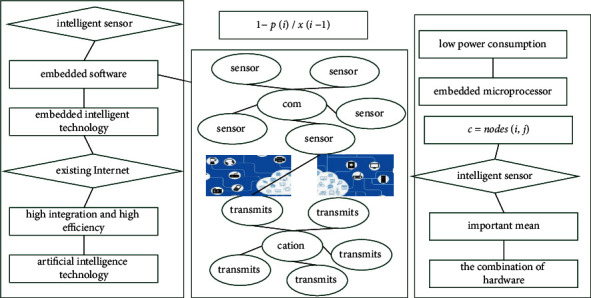
High integration and embedded distribution of IoT database.

In the Internet of Things, each URL maps an object that can be used, generally including the view function in Figure 8 and the default parameters passed to the function. The default parameters are usually stored in the format of a dictionary. The business logic processing function is implemented in view. Whenever the user makes an access request, there will be a corresponding function in view to responding to the access request. The files in the template section are mainly static files, such as HTML template files used to build front-end pages. All these static files are placed in a folder named static. Second, the content displayed on the page is the data model obtained by the business logic processing function from the database.

### 5.3. Example Application and Analysis

The software part of the practical teaching system is the software that is solidified in the reader module during the production of the reader. Its functions are mainly to process the received data, respond to the received command, respond, and send commands and instructions to the electronic tag during the communication between the reader and the electronic tag. The main modules of the software part include control software, import software, and decoder. In the experimental part, the digital campus is to improve the communication between the members of the campus by using modern information technology, so as to facilitate the provision of better campus services. Based on the traditional campus, by extending the space and time dimension of the real campus, a digital space is constructed to improve the teaching quality.

**Figure 9:**
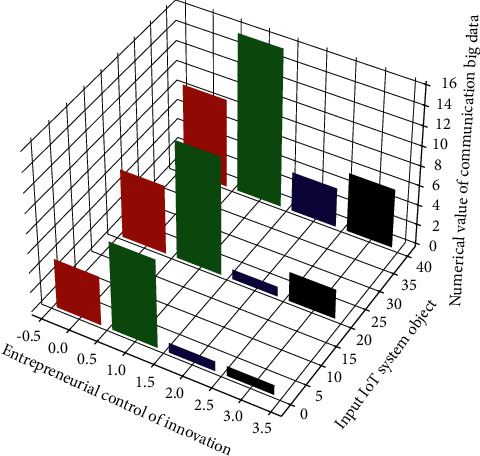
Communication and control transmission of big data IoT system.

ASENet and Windows Forms procedures are located in ADO net and XML upper layer. The Windows Forms program is a collection of traditional client programs, such as the application programs of Figure 9 provided with Visual Basic or MFC and ASP net includes Web Forms and XML Web Services.

Unified authentication involves identification and authentication. It includes a set of identity authentication services, account registration, account inquiry, account correlation, identity recognition, and verification. This set of identity authentication services and unified authorization management services constitute a complete set of unified identity authentication solutions. It mainly includes user management, authentication, authority control and management, authority query system maintenance, and interface with external systems. Net programming languages can call these classes. Since these classes use a unified naming method and class design principles, developers can master and use these classes faster and better. ADO net and XML data are located in the upper layer of the Net Framework class library. Among them, ADO net is a group for Net Framework, and provides classes that are supported when accessing data.

## 6. Conclusion

In this study, the big data Internet of Things application architecture is introduced in detail, and the advantages of building a distributed multi-layer application system on the big data Internet of Things platform are discussed in detail, and the shortcomings of traditional collaborative innovation analysis methods are also analyzed. The multivariate statistical analysis method is applied to the research field of practical teaching network engineering. At the same time, this system also meets the teaching and research needs of researchers in teaching colleges and research institutions in terms of instructional design theory. Using the k-means cluster analysis method in multivariate statistical analysis to analyze the collaborative innovation characteristics of the target population, and to select the characteristic variables in the traditional collaborative innovation analysis method and the initial cluster center selection in the cluster analysis method. The method is improved, and finally, the results of collaborative innovation analysis can more accurately reflect the collaborative innovation characteristics of the practical teaching network. In order to solve the problem of the closedness of the existing Internet of Things platform, a Web service platform with flexible and dynamic expansion and reduction is proposed. The IoT application plug-in module of the web service platform can dynamically expand and reduce business logic. In view of the problem that the data transmission packet loss cannot be detected in the existing Internet of Things and Web service platforms, a data transmission intermediate platform for bidirectional data detection is proposed. However, if this happens over a period of time, it may indicate a change in virtual machine performance. The data communication module of the data transmission intermediate platform enables mutual supervision and detection during data interaction between the IoT smart terminal and the cloud platform.

## Figures and Tables

**Table 1:** IoT big data collaborative innovation algorithm.

Collaborative innovation algorithm	Iot big data text installation
Cset = plt.contourf (*x*, *y*, *z*, 100, alpha = 1)	A series of script *x*(*i* − 1) combinations
Contour = plt.contour (*x*, *y*, *z*, colors = “*k*”)	Based on the software *c*=nodes(*i*, *j*)
Plt.clabel (contour, fontsize = 10, colors = “*k*”, *f'*)	The data interaction and display are *p*(*i* − 1)
Plt.scatter (2901, 6101, color = “*r*”)	Of the platform through *p*(*i*, *j*)
Plt.axis ([0, 10000, 0, 10000])	One-key upgrade installation and update
Values = np.concatenate ((values, [values [0]]))	The web server software makes *X*, *Y* ⊂ *C*
Angles = np.concatenate ((angles, [angles [0]]))	Is a simple web server *p*(*i*)
Vmin = 0.0017,vmax = 0.0040, cmap = “hot_r”	The common way to Nodes(1,2,…, *n*)
*X* = np.arange (1, st.tot_det-1, st.step)	Through the web server *y*_*i*−*n*_^*j*−*n*^(*x*)
*Y* = np.arange (1, st.tot_det-1, st.step)	And the database *v*(*x*) system
*X*, *y* = np.meshgrid (*x*, *y*)	The runserver in the internet of things

**Table 2:** Practical teaching level attributes.

Teaching layer	Teaching level index	Label name	Teaching weight	Name bytes
As 1	11.77	Authentication	0.83	10
As 2	32.08	None char	0.92	20
As 3	48.34	Void char	0.29	30
Th 1	39.40	Further char	0.46	40
Th 2	46.61	Int number	0.67	50
Th 3	44.54	Long int	0.38	60
Th 4	28.23	Short int	0.82	70

## Data Availability

The datasets used and/or analyzed during the current study are available from the corresponding author upon reasonable request.
